# Effects of Including Partially Destoned Olive Cake in Sheep Diet on Meat Quality and Salami Production

**DOI:** 10.3390/ani16020347

**Published:** 2026-01-22

**Authors:** Giuseppe Maniaci, Riccardo Gannuscio, Cristina Giosuè, Mahmood Ul Hassan, Gabriele Busetta, Elena Franciosi, Raimondo Gaglio, Massimo Todaro, Marco Alabiso

**Affiliations:** 1 Department of Agricultural, Food and Forestry Science (SAAF), Università di Palermo, Viale delle Scienze 13, 90128 Palermo, Italy; giuseppe.maniaci@unipa.it (G.M.); riccardo.gannuscio@unipa.it (R.G.); mahmoodul.hassan@unipa.it (M.U.H.); gabriele.busetta@unipa.it (G.B.); raimondo.gaglio@unipa.it (R.G.); massimo.todaro@unipa.it (M.T.); marco.alabiso@unipa.it (M.A.); 2 Institute of Marine Science, National Council of Research (ISMAR-CNR), c/o Campus Università di Palermo, Via Archirafi snc, 90123 Palermo, Italy; 3 Research and Innovation Centre, Fondazione Edmund Mach (FEM), Via E. Mach 1, 38098 San Michele all’Adige, Italy; elena.franciosi@fmach.it

**Keywords:** sheep meat, olive cake, nutritional quality, fatty acid profile, salami production, sustainable livestock production

## Abstract

The valorization of cull sheep and the incorporation of agro-industrial by-products into animal feeding represent effective approaches to enhancing the sustainability of small ruminant production systems. This study investigated the effects of dietary inclusion of 17% partially destoned olive cake (OC) in the concentrate fed to Valle del Belice ewes on carcass characteristics, as well as on meat and salami quality. A 14-week feeding trial was conducted on 124 animals allocated to a control (CTR) and an experimental (EXP) group, balanced for parity, days in milk, and daily milk yield. At the end of the trial, five animals per group were slaughtered and their meat was processed into three types of salami: 100% sheep meat (SM), 90% sheep meat with 10% beef heifer brisket (HB), and 90% sheep meat with 10% pork backfat (PB). Meat and salami were evaluated for chemical composition, fatty acid profile, polyphenol content, antioxidant capacity, lipid oxidation, microbiological status, textural properties, and sensory characteristics. Dietary OC supplementation resulted in increased carcass weight, separable fat, intramuscular fat content, and monounsaturated fatty acids—particularly oleic acid—along with higher polyphenol levels and antioxidant activity. Salami produced from OC-fed ewes exhibited reduced weight loss during ripening, lower lipid oxidation, an improved MUFA/SFA ratio, and satisfactory sensory attributes. Microbiological analyses indicated a dominance of lactic acid bacteria and coagulase-negative staphylococci, with no pathogenic microorganisms detected. Overall, the inclusion of olive cake in the diet enhanced meat and processed product quality, supporting the valorization of olive oil by-products within circular economy frameworks.

## Simple Summary

At the end of their productive lifespan, sheep often yield meat of limited commercial value, while substantial quantities of by-products from olive oil production remain underutilized. The present study investigated the effects of incorporating processed olive oil residues into sheep diets on meat quality and derived products, with the dual aim of valorizing an agro-industrial by-product and improving product sustainability. Over a 14-week feeding trial, two groups of sheep were administered isoenergetic concentrate diets, one of which included destoned olive cake. Following the experimental period, meat from each group was processed into different types of salami. Sheep receiving the destoned olive cake diet exhibited increased carcass weight and meat characterized by a more favorable lipid profile, including higher levels of beneficial fatty acids and bioactive plant-derived compounds with recognized antioxidant properties. Salami produced from this meat showed reduced weight loss during ripening, lower lipid oxidation, and satisfactory sensory attributes, without evidence of microbial safety concerns. Overall, these findings indicate that the inclusion of destoned olive cake in sheep feed can improve meat and processed product quality while promoting the sustainable reuse of olive oil by-products, offering benefits for both livestock production systems and environmental management.

## 1. Introduction

Sheep meat, commonly referred to as lamb or mutton, is a nutrient-rich animal-derived food that is widely available in regions with substantial sheep populations. The meat obtained from Ovis aries is consumed by millions of people worldwide and is likely eaten to some extent in nearly every country [1]. Although this category of livestock is traditionally not regarded as a major source of premium-quality meat, the effective utilization of meat from cull ruminants may constitute a valuable approach to improving both the economic viability and environmental sustainability of livestock production systems [2,3].

The chemical, nutritional, and sensory properties of sheep meat are strongly influenced by feeding strategies [4,5], as well as by the processing technologies applied during meat transformation [6]. In this context, dietary manipulation represents a key tool for improving meat quality, primarily through modifications of the fatty acid profile—such as increasing the deposition of health-promoting polyunsaturated fatty acids—and through the modulation of antioxidant compound concentrations in muscle tissue [4,5,6].

Fresh meat obtained from end-of-career cattle, ewes, and goats is generally characterized by lower tenderness and reduced market appeal compared with that from younger animals; however, its compositional traits—often including higher intramuscular fat content and a more pronounced flavor profile—make it well suited for processing into cured meat products such as salami, bresaola, and mortadella [7,8,9]. Processing technologies can simultaneously enhance sensory attributes, decrease microbial loads, and extend shelf life, thereby converting a low-value raw material into traditional or innovative high-value products. These improvements are primarily achieved through two main technological pathways: dry-curing and extended ripening, during which reductions in water activity (aw) and increased proteolytic and lipolytic activities contribute to improved tenderness and flavor complexity; and targeted thermal treatments, which ensure microbial inactivation and further support product safety and quality [10,11,12,13]. Moreover, the valorization of end-of-career animals through meat processing aligns with circular economy principles, promoting more efficient resource use and sustainability within the meat production chain [3,14].

Globally, sheep meat is traditionally employed in the production of a wide range of fermented and cured sausages, either as a sole raw material or in combination with other meats such as beef or pork, which can enhance sensory attributes, particularly flavor and textural properties [11]. Compared with pork, sheep meat is inherently leaner, a characteristic that may result in lower processing yields and reduced juiciness in salami-type products [15]. To overcome these technological constraints, several formulation strategies have been investigated. The inclusion of other meat sources or fat ingredients, such as beef meat or pork backfat, has been shown to improve juiciness, texture, structural integrity, and shelf life without adversely affecting nutritional quality [11,15]. Nevertheless, the use of pork-derived components limits product acceptability among certain consumer groups, notably Muslim consumers, for whom pork-free formulations are required to comply with Halal dietary standards [16]. Consequently, the development of products based on non-pork raw materials, including sheep meat, has gained increasing commercial relevance, in line with consumer demand for formulations with strictly controlled ingredient lists and the growing market trend toward inclusive, clean-label meat products that exclude common allergens or ingredients restricting consumption due to religious or lifestyle considerations [17]. Recent studies indicate that the substitution of pork backfat with alternative lipid sources, such as vegetable fats, may facilitate the production of healthier and culturally acceptable meat products, while achieving an optimal balance among sensory quality, lipid profile, and shelf life [18,19]. In parallel, the incorporation of functional ingredients, including olive-derived extracts and other natural antioxidant sources, into sheep meat salami has been shown to enhance oxidative stability, nutritional value, and the content of bioactive compounds [20].

In recent years, the incorporation of agro-industrial by-products into sheep diets, including olive oil processing residues such as olive cake, has attracted increasing scientific and commercial interest [21,22,23]. Olive cake, a major by-product of the olive oil industry, is characterized by a high content of polyphenols, antioxidant compounds, and dietary fiber [14]. These bioactive components have been shown to contribute to improved animal welfare by mitigating physiological stress responses, while concurrently exerting positive effects on the chemical and nutritional quality of animal-derived products, including milk and meat [14,24,25]. In particular, dietary supplementation with olive cake has been associated with reduced lipid oxidation in meat and enhanced oxidative stability, both of which are critical determinants of sensory quality, color stability, and shelf life [14,24]. Furthermore, the inclusion of olive cake in livestock diets has been reported to favorably modify meat lipid composition by increasing monounsaturated fatty acid content, thereby improving the overall nutritional profile and influencing key sensory attributes such as flavor and textural characteristics [24].

From both economic and environmental standpoints, the inclusion of olive oil by-products in sheep feeding strategies is consistent with circular economy principles, as it promotes the reduction of agricultural waste and decreases reliance on external feed resources, thereby contributing to a lower carbon footprint associated with feed and meat production systems [14,26,27]. Consequently, the integration of olive cake by-products into livestock diets may enhance the value chains of both the olive oil and sheep meat sectors, supporting their overall sustainability by reducing feeding costs, optimizing resource efficiency, and mitigating environmental impacts. This approach ultimately contributes to the development of more efficient and sustainable livestock production systems [28].

Within this framework, the present study aimed to investigate the effects of dietary supplementation with partially destoned olive cake by-products on the quality of sheep meat and derived salami. Specifically, the study evaluated the influence of different feeding strategies on the chemical composition, nutritional profile, and sensory characteristics of both fresh meat and processed products.

## 2. Materials and Methods

### 2.1. Animals, Experimental Design, and Diets

The experiment was conducted in accordance with Italian Legislative Decree No. 26/2014 [29], implementing Directive 2010/63/EU [30] on Animal Welfare and Good Clinical Practice, and received the approval of the Animal Welfare Body (OPBA) of the University of Palermo (protocol number: UNPA-CLE-Prot.117.247, 12 July 2024).

The trial was carried out for 14 weeks (from April to June 2024), including 14 days for the adaptation period and 12 weeks for the sampling, and involved 124 Valle del Belice lactating dairy ewes raised in a farm located at 736 m above sea level (a.s.l.) in Castronovo di Sicilia (Italy—latitude 37°39′22″ N–13°33′28″ E), in the typical production area for this breed.

The 124 ewes were divided into two groups balanced to their parity (≥3rd lambing), days in milk (60 ± 5), and daily milk yield (1.48 ± 0.4 kg/d). The animals in each group were fed on grazing pasture and one of the following experimental concentrates: (i) a concentrate without partially destoned olive cake supplementation (concentrate CTR); (ii) a concentrate containing 17% partially destoned olive cake (OC) (as feed basis) supplementation (concentrate EXP). The OC used was derived from a two-stage olive oil extraction process. The process involves initial stabilization of the wet pomace with hydrochloric acid (E 507), followed by 180 days of storage. After centrifugation and sun-drying (to 8–10% moisture), the material is mechanically crushed and subjected to a cyclone separation system to obtain partial pitting. The partially pitted product was selected because removing the highly lignified pits substantially reduces the lignin content, thus increasing digestibility, and concentrates the residual pulp, with a higher concentration of fatty acids and polyphenols in the final product.

The dietary ingredients used to formulate both experimental concentrates are given in Table 1, while the chemical composition of feeds is reported in Table 2. The concentrates were individually offered to every animal in each group once a day at 4:00 p.m., at a rate of 0.5 kg/ewe (as feed basis), and were entirely consumed by all the animals of both groups. Water was offered ad libitum to both groups. The concentrates used in this study were formulated to be isoenergetic. Throughout the experimental period, the general health conditions of the animals were monitored and remained optimal.

At the end of the 14-week trial, 10 randomly chosen ewes (5 CTR and 5 EXP) were slaughtered at an EU-approved abattoir following standard handling procedures and in compliance with EU Regulation EC No. 1099/2009 [31] on the protection of animals at the time of slaughter. The meat from their entire carcasses was used for salami produced at the “Amato salumi” company in Camporeale (Sicily, Italy).

### 2.2. Feed Sampling and Analysis

Every two weeks, the control and experimental concentrates were sampled, and the sheep were observed during grazing. Their selection of forage resources was recorded and sampled, with plant parts manually collected (hand-plucked) to reconstruct the diet according to the method described by Maniaci et al. [32]. In brief, the behaviour of sheep in the pasture was observed. There were two observers per experimental group, positioned in the pasture (using binoculars when necessary). Observations of behaviour were made every 15 min over an 8 h period (from 8:00 h to 16:00 h) using scan sampling, which consisted of a quick observation of the animal’s activity (grazing, drinking, ruminating, walking, idling) recording the type of quantitative-qualitative selection performed. The concentrate provided in the stable was completely consumed by the animals, while pasture intake was not evaluated, therefore it was not possible to calculate the overall nutrient intake.

The pasture samples, placed in sterile containers, were refrigerated at 8 °C during transfer to the laboratory, where they were homogenized and stored at −20 °C. The pasture samples were freeze-dried before the analysis. All feeds were analyzed for dry matter (DM—method 967.03), crude protein (CP, N × 6.25—method 988.05), ether extract (EE—method 920.29), and ash (method 942.05), following AOAC methods [33]. Neutral detergent fiber organic matter (NDFom), acid detergent fiber organic matter (ADFom), and acid detergent lignin (ADL) were determined according to Van Soest et al. [34]. Non-fiber carbohydrates (NFCs) were calculated as NFC = 100 − [CP + EE + Ash + NDFom] [32].

Fatty acids (FAs) were extracted from all feed samples using the method developed by O’Fallon et al. [35], with C23:0 used as the internal standard (Sigma-Aldrich, Milan, Italy). Each sample (1 µL) was injected using an autosampler into an HP 6890 gas chromatography system equipped with a flame ionization detector (Agilent Technologies Inc., Santa Clara, CA, USA). Individual FAs were separated and identified as described by Alabiso et al. [36].

The total phenolic content was determined in all the samples. Extraction was performed according to the procedure described by López-Andrés et al. [5]. The total polyphenol concentration was measured using the Folin–Ciocalteu colorimetric method, as described by López-Andrés et al. [5]. Absorbance was read at 725 nm using a HACH DR/4000U spectrophotometer (HACH, Loveland, CO, USA) against a blank containing all the reagents but not the sample extract. Aqueous solutions of gallic acid at various concentrations (0–1 mg/mL) were used to construct the calibration curve (R^2^ = 0.99). The results are expressed as grams of gallic acid equivalents (GAE) per kilogram of dry matter (DM).

### 2.3. Manufacturing Processes and Analytical Evaluation of Meat and Salami Products

After slaughter, the carcasses were stored in a refrigerated chamber at 4 °C for an aging period of 4 days. Subsequently, the carcasses were sectioned and deboned (day 0), separating the meat from bones and separable fat.

Live weight was recorded prior to slaughter, and for each carcass, hot and cold carcass weights were measured, along with the weights of the three fractions obtained after deboning. For each carcass, a section of the *Longissimus dorsi* muscle, between the 8th thoracic vertebra and the 1st lumbar vertebra cleaned of external fat, was collected.

Meat from each group (CTR, EXP), after being cleaned of subcutaneous fat, was ground using a 6 mm plate and divided into three portions (batch a, b and c), each batch was utilized to prepare the following mixtures: 100% sheep meat (SM), 90% SM + 10% heifer brisket (HB), and 90% SM + 10% pork backfat (PB). Ground meat samples were collected from both sheep groups, along with samples of heifer brisket and pork back, which were purchased commercially and originated from the same production batch.

All mixtures (SM, HB, and PB) were supplemented with curing mix (15 g/kg) (sucrose, dextrose and maltodextrin, 95%; sodium ascorbate, 4%; sodium nitrate and potassium nitrate, 1%), salt (27 g/kg), ground black pepper (4 g/kg), and white wine (12 mL/kg) in which minced garlic (58.5 g/L) had been macerated for 12 h and subsequently filtered.

Each mixture was thoroughly blended and stuffed into natural casings, resulting in salami with an average weight of 460 ± 25 g, which was aged for 32 days in a temperature- and humidity-controlled chamber.

The salami were sampled on day 0 (immediately after mixing, referred to as raw mixture) and days 10, 21, and 32 (end of the ripening process).

All samples were placed in sterile vacuum-sealed containers, immediately refrigerated, and transported at 8 °C to the laboratory. They were homogenized using a stomacher (LAB Blender 400, Seward Medical, London, UK) for 2 min at maximum speed and then frozen at −20 °C and freeze-dried for further analysis (SCANVAC Coolsafe 55-9, Labogene Aps, Lynge, Denmark).

#### 2.3.1. Physical and Chemical Parameters

At slaughter, the pH and color were assessed in both hot and cold states at the *Longissimus lumborum* level, between the 3rd lumbar vertebra and the 5th lumbar vertebra, on the right for the hot state and on the left for the cold state. Moreover, the color of perirenal fat was also recorded in the hot state. The pH was detected with a digital pH meter (Thermo Orion 710 A +, Cambridgeshire, UK), equipped with a penetration probe. The color was determined using a Chroma Meter (CR-300; Minolta, Osaka, Japan). The results are expressed as lightness (L*), redness (a*), and yellowness (b*) according to the CIE Lab* color system [37]. Chroma [C = (a*2 + b*2)^0.5^] and hue angle [H = (arctg b*/a*)] were calculated using the a* and b* values.

Raw materials (sheep, heifer brisket and pork backfat) and salami samples (0, 10, 21, and 32 days) were analyzed for pH using a pH meter (BASIC 20+; Crison Instrument S.A., Barcelona, Spain) using 10 g of sample diluted with 90mL distilled water and homogenized using a stomacher (LAB Blender 400; Seward Medical, London, UK) at maximum speed for 2 min. Salami samples were peeled prior to dilution.

Salami weight loss was expressed as the difference from the initial weight.

Moreover, the same samples were analyzed for color, using the same procedure described for *Longissimus lumborum.*

On the *Longissimus dorsi*, sampled after deboning, the Warner–Bratzler shear force (WBS) was measured with an Instron Universal Testing Machine (Instron tester 5564, Trezzano sul Naviglio, Milan, Italy) equipped with a Warner–Bratzler device; for each muscle, 5 samples with a 10 × 10 mm cross-section and 25 mm length were detected.

To evaluate the texture of the salami samples at the end of ripening, 25 mm thick transverse slices were cut, peeled, and subjected to a 50 kg load cell force using a universal testing machine (Instron 5564; Trezzano sul Naviglio, Milan, Italy). The results are expressed as maximum compression strength (Hardness, N/mm^2^).

The chemical composition of all the samples (*Longissimus dorsi* after deboning; raw materials; and salami at 0, 10, 21, and 32 days) was determined according to standard AOAC methods [31]: dry matter (DM—method 967.03), crude protein (CP, N × 6.25—method 988.05), ether extract (EE—method 920.29), and ash (method 942.05). Non-protein nitrogen (NPN) was determined in the salami on days 0 and 32 by analyzing the supernatant after protein precipitation with 5% trichloroacetic acid. The proteolysis index (PI) was calculated as the percentage ratio of NPN to total nitrogen (TN) [38]. All analyses were performed in triplicate for each sample at each time point.

#### 2.3.2. Fatty Acid Composition

The fatty acid (FA) profile was determined for the raw materials and salami at the end of ripening according to the method developed by O’Fallon et al. [35] using the same procedure adopted for feed.

The atherogenic index (AI) and thrombogenic index (TI) were calculated as reported by Ulbritch and Southgate [39]:

(1) $$\mathrm{AI}\;=\;\frac{C12:0\;+\;\left(4*C14:0\right)\;+\;C16:0}{PUFA+MUFA}$$

(2) $$\mathrm{TI}\;=\;\frac{C14:0\;+\;C16:0\;+\;C18:0}{\left(0.5\cdot \Sigma MUFA)\;+\;(0.5\cdot \Sigma PUFA\omega 6)\;+\;(3\cdot \Sigma PUFA\omega 3)\;+\;(\Sigma PUFA\omega 3/\Sigma PUFA\omega 6\right)}$$

#### 2.3.3. Polyphenols, Antioxidant Capacity, and Lipid Oxidation

The total polyphenols and Trolox equivalent antioxidant capacity (TEAC) of the raw materials and salami samples were determined to analyze their antioxidant properties, as well as their oxidative stability.

The total polyphenols were determined by the Folin–Ciocalteau colorimetric method [5] using the same procedure adopted for feed, while the TEAC was determined with a discoloration test measuring the radical scavenging ability of samples using the ABTS radical cation (ABTS+) and Trolox as the standard [40]. A Trolox solution in PBS was used to develop a calibration curve (R^2^ = 0.99), and the results are expressed as mmol Trolox/kg DM.

The oxidative stability of meat fat was evaluated by determining the peroxide value (POV) (g gallic acid equivalent (GAE)/kg DM) as the index of primary lipid oxidation [41], and the thiobarbituric acid-reactive substances (TBARs) as products of secondary lipid oxidation. The TBARs are expressed as µg of malonylaldehyde (MDA)/kg DM and were quantified using a calibration curve (R^2^ = 0.99) obtained with 1,1,3,3-tetramethoxypropane solutions at different concentrations.

#### 2.3.4. Sensory Analysis

The sensory profiling of the aged salami was carried out by the National Organization of Salumi Tasters (ONAS), using the official ONAS evaluation form ‘Salumi Crudi Macinati’, and involved qualitative and quantitative evaluation of the salami using the senses (sight, smell, taste, and touch), concluding with an overall assessment of the taste–olfactory balance. The following descriptors were included in the analysis: external examination, visual examination, odor, odor intensity, taste, aroma, persistence, consistency, chewiness, and sensory balance.

The qualitative evaluations assigned a numerical value on a scale of 0 (extremely low) to 10 (extremely high). Each sample was evaluated in all its parts, externally by looking and touching the piece, and internally by observing and tasting the slice.

The descriptive panel consisted of sixteen trained assessors (8 females and 8 males, aged between 25 and 65 years), all of whom regularly perform sensory evaluations and have experience in assessing meat products (including fermented products), as well as familiarity with sensory methodology and technical aspects.

The panelists underwent preliminary training sessions aimed at reaching consensus on the sensory descriptors and the use of the intensity scale.

#### 2.3.5. Culture-Dependent Microbiological Assessment of Salami Productions

Ten grams of each raw material and salami sample, collected at different stages of ripening along the ovine salami production chain, were immediately transferred under refrigerated conditions to the Laboratory of Agricultural, Food and Environmental Microbiology at the University of Palermo. There, the samples were homogenized in 90 mL of saline-peptone solution [0.8% (*w*/*v*) NaCl and 0.1% (*w*/*v*) bacteriological peptone] using a stomacher machine (BagMixer^®^ 400, Interscience, Saint-Nom-la-Bretèche, France). The resulting suspensions were used to prepare serial decimal dilutions, which were plated on agar media to support the growth of microbial groups relevant to meat and fermented meat production. Specifically, total mesophilic microorganisms (TMM) were enumerated on Plate Count Agar (PCA) after incubation at 30 °C for 72 h. Mesophilic lactic acid bacteria (LAB) were isolated on de Man-Rogosa-Sharpe (MRS) agar supplemented with cycloheximide (0.1 g/L) and incubated at 30 °C for 48 h. The presence of coagulase-positive staphylococci (CPS) and coagulase-negative staphylococci (CNS) was investigated on Baird-Parker (BP) agar supplemented with rabbit plasma fibrinogen (RPF). Yeasts were enumerated on Dichloran–Rose Bengal Chloramphenicol (DRBC) agar incubated at 25 °C for 5 days, while molds were enumerated on Potato Dextrose Agar (PDA) supplemented with 0.1 g/L chloramphenicol to avoid bacterial growth under aerobic incubation at 25 °C for 7 days. Regarding hygiene indicator microorganisms, *Escherichia coli* was detected on Hektoen Enteric Agar (HEA), while the members of the Enterobacteriaceae family were determined on Violet Red Bile Glucose Agar (VRBGA). In addition, *Salmonella* spp. and *Listeria monocytogenes* were detected following the standardized procedures described in ISO 6579-1 [42] and ISO 11290-1 [43], respectively. All media were supplied by Lickson (Vicari, Italy). Microbiological counts were performed in duplicate for each sample and at each sampling time.

#### 2.3.6. Culture-Independent Profiling of the Bacterial Community of Salami

Genomic DNA was extracted from samples using the DNeasy PowerFood Microbial Kit (QIAGEN, Hilden, Germany) following the manufacturer’s instructions to ensure both yield and integrity. The DNA quality was checked by agarose gel electrophoresis to verify fragment integrity, and by UV/Vis spectrophotometry to assess purity and concentration.

The V3–V4 hypervariable regions of the bacterial 16S rRNA gene were amplified with the primer pair 341F (5′-CCTACGGGNGGCWGCAG-3′) and 806R (5′-GACTACNVGGGTWTCTAATCC-3′), as previously reported by Busetta et al. [44]. PCR products were purified using AMPure XP magnetic beads (Beckman Coulter, Brea, CA, USA), and sequencing libraries were prepared for analysis on the Illumina^®^ MiSeq platform (Illumina Inc., San Diego, CA, USA). Sequence data were processed through the DADA2 pipeline [45], which included quality filtering, trimming of low-quality bases, denoising, and merging of paired-end reads. Taxonomic assignment was performed against the Greengenes database (version 13_8, 99%) to define Operational Taxonomic Units (OTUs). The resulting FASTQ files have been deposited in the NCBI Sequence Read Archive (SRA) under the accession number PRJNA1338951.

### 2.4. Statistical Analysis

The data of the animals and carcass characteristics were statistically analyzed using the SAS 9.2 software [46], using a generalized linear model that included the fixed effects of diet (2 levels; CTR and EXP). The results are reported as least median of squares (LSM), and differences between means were tested by Tukey’s *t*-test. Statistical significance was attributed to *p* values <0.05.

The data of the salami were statistically analyzed using a generalized linear model that included the fixed effects of diet (2 levels; CTR and EXP) [46], mixture (3 levels; SM, HB, and PB), and the interaction diet × mixture. The results are reported as LSM, and differences between means were tested by Tukey’s *t*-test. Statistical significance was attributed to *p* values <0.05.

For each culture medium and each sampling time, microbial data (log CFU/g) were analyzed using a generalized linear model [46] that included the fixed effects of diet (2 levels; CTR and EXP), mixture (3 levels; SM, HB, and PB), and the interaction diet x mixture. The results are reported as LSM, and differences between means were tested by Tukey’s *t*-test. Statistical significance was attributed to *p* values <0.05.

## 3. Results

### 3.1. Chemical and Physical Composition of Feed

The chemical composition of the feeds used in the experimental trial is reported in Table 2. 

**Table 2:** Chemical composition of feeds (means ± DS).

Items	Concentrate CTR	Concentrate EXP	Pasture	Olive Cake
Dry Matter, kg	88.72 ± 0.32	89.20 ±0.24	21.42 ± 8.00	93.91 ± 0.22
Crude Protein, % of DM	19.06 ± 0.48	20.97 ± 0.28	14.92 ± 3.54	11.50 ± 0.34
Ether extract, % of DM	2.75 ± 0.08	5.68 ± 0.39	2.28 ± 0.32	21.70 ± 0.55
NDFom, % of DM	32.19 ± 2.58	36.48 ± 1.14	46.06 ± 9.47	62.27 ± 1.10
ADFom, % of DM	15.23 ± 1.74	16.70 ± 1.06	28.90 ± 6.02	48.78 ± 0.54
ADL, % of DM	2.04 ± 0.08	3.88 ± 0.05	4.77 ± 1.36	22.08 ± 1.70
Ash, % of DM	5.76 ± 0.43	4.97 ± 0.18	2.98 ± 0.57	3.40 ± 0.65
NFC	40.25 ± 2.99	31.89 ± 1.38	24.43 ± 5.95	1.14 ± 0.12
NE_L_ (kcal/kg FM) [47]	1589 ± 27.3	1583 ± 23.6	--	--
NE_L_ (UFL/kg FM) [47]	0.90 ± 0.03	0.90 ± 0.02	--	--
C16:0 (% FA tot)	18.65 ± 0.92	17.78 ± 0.84	18.37 ± 1.45	19.14 ± 1.07
C18:0 (% FA tot)	1.58 ± 0.18	2.23 ± 0.27	2.23 ± 0.53	2.91 ± 0.25
C18:1 *cis n*-9 OA (% FA tot)	19.27 ± 1.05	35.55 ± 1.27	10.63 ± 4.50	55.56 ± 1.29
C18:2 *cis n*-6 LA (% FA tot)	51.49 ± 3.17	35.67 ± 2.41	24.23 ± 8.36	12.36 ± 1.23
C18:3 *cis n*-3 ALA (% FA tot)	5.21 ± 0.43	3.13 ± 0.27	35.38 ± 10.61	0.76 ± 0.09
SFA (% FA tot)	20.98 ± 0.83	20.84 ± 0.77	23.92 ± 2.38	22.94 ± 1.13
MUFA (% FA tot)	21.54 ± 1.12	38.36 ± 1.57	12.73 ± 5.27	59.87 ± 1.31
PUFA (% FA tot)	57.48 ± 2.26	40.80 ± 2.17	63.35 ± 7.52	17.20 ± 1.49
Polyphenols mg GAE/g DM	3.79 ± 0.47	5.60 ± 0.06	14.34 ± 3.21	7.52 ± 0.44

FM = Fresh Matter; CTR = control; EXP = experimental; NDFom = NDF organic matter. ADFom = ADF organic matter; NFC = 100 − (CP + ether extract + ash + NDFom). NE_L_ = Net Energy for lactation; OA = oleic acid; LA = linoleic acid; ALA = α-linolenic acid; SFA: Saturated fatty acids; MUFA: Monounsaturated Fatty Acids; PUFA: Polyunsaturated Fatty Acids; GAE = gallic acid equivalent.

Inclusion of partially destoned olive cake (OC) in the experimental (EXP) concentrate resulted in a composition distinctly different from that of the control (CTR) diet. Specifically, the EXP concentrate contained higher crude protein (CP; 20.97% vs. 19.06%) and greater ether extract (EE; 5.68% vs. 2.75%), reflecting the naturally high lipid content of the OC by-product (21.70%). Likewise, the neutral detergent fiber (NDFom) and acid detergent lignin (ADL) fractions were increased in the EXP concentrate. The fatty acid profile of the EXP concentrate also displayed notable nutritional differences, with elevated monounsaturated fatty acids (MUFA; 38.36% vs. 21.54%) and reduced polyunsaturated fatty acids (PUFA; 40.80% vs. 57.48%). Furthermore, the EXP concentrate exhibited a higher total polyphenol content than the CTR diet (5.60 vs. 3.79 mg GAE/g DM). Pasture composition was characterized by a high proportion of PUFAs (63.35%), primarily α-linolenic acid (35.38%), and a relatively elevated polyphenol content (14.34 mg GAE/g DM).

### 3.2. Carcass Traits and Meat Composition

The inclusion of olive cake (OC) in ewe diets resulted in several notable changes in carcass traits and meat quality (Table 3). Both hot and cold carcass weights were significantly higher in the EXP group compared with CTR, although no significant differences were observed in hot or cold pH values, with a trend toward lower pH in EXP sheep. Carcass composition analysis revealed that EXP animals had a lower proportion of bone (*p* = 0.016) and a higher proportion of separable fat (*p* = 0.030), showing a trend toward an increased proportion of meat, although this was not statistically significant.

Colorimetric analysis of perirenal fat and Longissimus dorsi (LD) muscle demonstrated significant differences between groups. In perirenal fat, EXP animals exhibited lower b* (yellowness; *p* = 0.046) and chroma values (*p* = 0.019). In LD muscle, CTR animals showed significantly higher b* and hue values in both hot (*p* = 0.012 and *p* = 0.017, respectively) and cold samples (*p* = 0.004 for both). Shear force analysis indicated no significant differences in tenderness between CTR and EXP meat.

Regarding chemical composition, intramuscular fat content was significantly higher in the EXP group (*p* = 0.003), whereas moisture content was significantly lower compared with CTR (*p* = 0.012).

The fatty acid composition (% of total FA) of the *Longissimus dorsi* muscle is presented in Table 4. Inclusion of olive cake in the EXP diet resulted in a significant increase in monounsaturated fatty acids (MUFA; *p* = 0.039), primarily due to higher oleic acid (C18:1 c9; OA) content (*p* = 0.046). Stearic acid (C18:0) was reduced in EXP samples. Furthermore, levels of C17:1 (*p* = 0.010) and C17:0 anteiso (*p* = 0.019) were higher in the EXP group, whereas other C18:1 isomers were significantly lower (*p* = 0.007). Total fatty acid content was also significantly greater in EXP samples compared with CTR (*p* = 0.034).

### 3.3. Physical and Chemical Composition of Raw Materials Used in Salami Production

The compositional characteristics of the raw materials used for salami production are summarized in Table 5. pH values varied among meat sources, ranging from 5.58 in EXP sheep meat to 6.10 in heifer brisket, with EXP sheep meat exhibiting a lower pH than CTR. Moisture content was highest in sheep meat (CTR: 71.40%; EXP: 67.43%), followed by beef (50.78%), and lowest in pork lard (20.66%), with EXP sheep meat showing reduced moisture compared to CTR. In contrast, fat content exhibited an inverse trend, being highest in pork lard (72.63%), followed by beef (34.32%), EXP sheep meat (13.01%), and CTR sheep meat (8.76%). Ash content was higher in CTR sheep meat (1.34%), intermediate in EXP sheep meat and beef (~1.00%), and lowest in pork lard (0.34%).

Polyphenol content and antioxidant capacity were greater in EXP sheep meat than in CTR (1.64 vs. 1.51 mg GAE/g DM and 13.05 vs. 11.42 mmol Trolox/kg DM, respectively). Regarding oxidative stability, the peroxide value (POV) was lowest in heifer brisket (1.61 mEq O_2_/kg fat), slightly lower in EXP than CTR sheep meat (2.24 vs. 2.50 mEq O_2_/kg fat), and intermediate in pork lard (2.02 mEq O_2_/kg fat). Similarly, TBARS values were lowest in both CTR and EXP sheep meat (~0.07 mg MDA/kg tissue), whereas higher values were recorded in pork lard (0.14 mg MDA/kg tissue).

The fatty acid composition of the raw materials used for salami production is presented in Table 6. The four matrices—sheep CTR, sheep EXP, heifer brisket and pork lard—displayed distinct lipid profiles. Ovine meats exhibited saturated fatty acids (SFA) between 46% and 48% and monounsaturated fatty acids (MUFA) between 45% and 47%, with oleic acid (C18:1 c9) as the predominant MUFA. Polyunsaturated fatty acids (PUFA) were relatively low in both sheep groups (~5–6%). In EXP sheep meat, a modest increase in MUFA (47.5% vs. 45.4%) and oleic acid (35.7% vs. 34.2%) was observed, accompanied by a decrease in PUFA (4.94% vs. 5.94%) and linoleic acid (2.02% vs. 3.19%). Moreover, EXP meat showed an improved n-3/n-6 ratio and slight reductions in atherogenic (AI) and thrombogenic (TI) indices. In contrast, heifer brisket was characterized by a higher SFA content (53.4%) and less favorable health indices, whereas pork lard contained the highest PUFA proportion (21.9%), largely due to linoleic acid (19.76%).

### 3.4. Physical and Chemical Composition of Salami

The physical characteristics of salami at the end of ripening are reported in Table 7, highlighting the effects of dietary treatment (G: CTR vs. EXP) and meat mixture (M: SM, HB, PB) on technological and structural properties. Color parameters showed significant variation due to the meat mixture, with the SM mixture exhibiting higher yellowness (b*; *p* = 0.0029) and hue angle (*p* = 0.0403) compared with other formulations. A trend toward increased b* values was observed in EXP salami, except for PB samples.

Weight loss (%) during ripening was significantly lower in salami prepared from EXP meat (*p* = 0.001), and this reduction was consistent across all meat mixtures. Meat mixture also had a significant effect on hardness (N/mm^2^), with PB salami exhibiting the lowest compression force (*p* < 0.001), followed by HB and SM formulations.

The chemical composition of salami at the end of ripening is presented in Table 8, showing significant effects of dietary treatment, meat mixture, and their interaction. Across all formulations, salami produced from EXP animals exhibited significantly lower moisture content compared with CTR counterparts (*p* < 0.002) and reduced ash content (*p* < 0.001). Diet also had a pronounced effect on protein and fat content, with EXP salami showing lower protein and higher fat percentages across all mixtures (*p* < 0.001). Ripening index (PI) values did not differ significantly among groups, ranging from 11.9% to 12.7%.

Polyphenol content (mg GAE/g DM) was slightly higher in EXP salami, particularly in the SM formulation (*p* = 0.003), and antioxidant capacity (TEAC, mmol Trolox/kg DM) tended to be greater in EXP salami, especially in SM (*p* = 0.046). Lipid oxidation parameters were consistently improved in EXP salami, with lower peroxide values (POV; mEq O_2_/kg fat; *p* < 0.001) and reduced TBARS (mg MDA/kg), particularly in SM and HB formulations.

### 3.5. Fatty Acid Profile of Salami

The fatty acid composition (% of total FA) of salami at the end of ripening is presented in Table 9. The fatty acid profile of the final products reflected both the meat mixture and the dietary treatment of the sheep. Across all formulations, SM EXP salami exhibited lower saturated fatty acid (SFA) content compared with SM CTR (*p* = 0.039) and higher monounsaturated fatty acids (MUFA; *p* = 0.019), primarily due to increased oleic acid (C18:1 c9; OA) levels (*p* = 0.043). This was accompanied by reduced atherogenic (AI; *p* = 0.047) and thrombogenic (TI; *p* = 0.019) indices in SM and HB EXP salami. Overall, EXP salami showed lower C16:0 and higher C18:1 c9 proportions.

In contrast, the inclusion of pork backfat significantly increased polyunsaturated fatty acids (PUFA; *p* < 0.0001) and decreased the n-3/n-6 ratio (*p* = 0.043), primarily due to elevated linoleic acid (C18:2 n−6; *p* < 0.0001). Incorporation of heifer brisket had minimal impact on the overall fatty acid profile relative to pure ovine formulations, with EXP samples exhibiting similar SFA reductions and slight MUFA increases.

### 3.6. Sensory Profile of Salami

**Figure 1:**
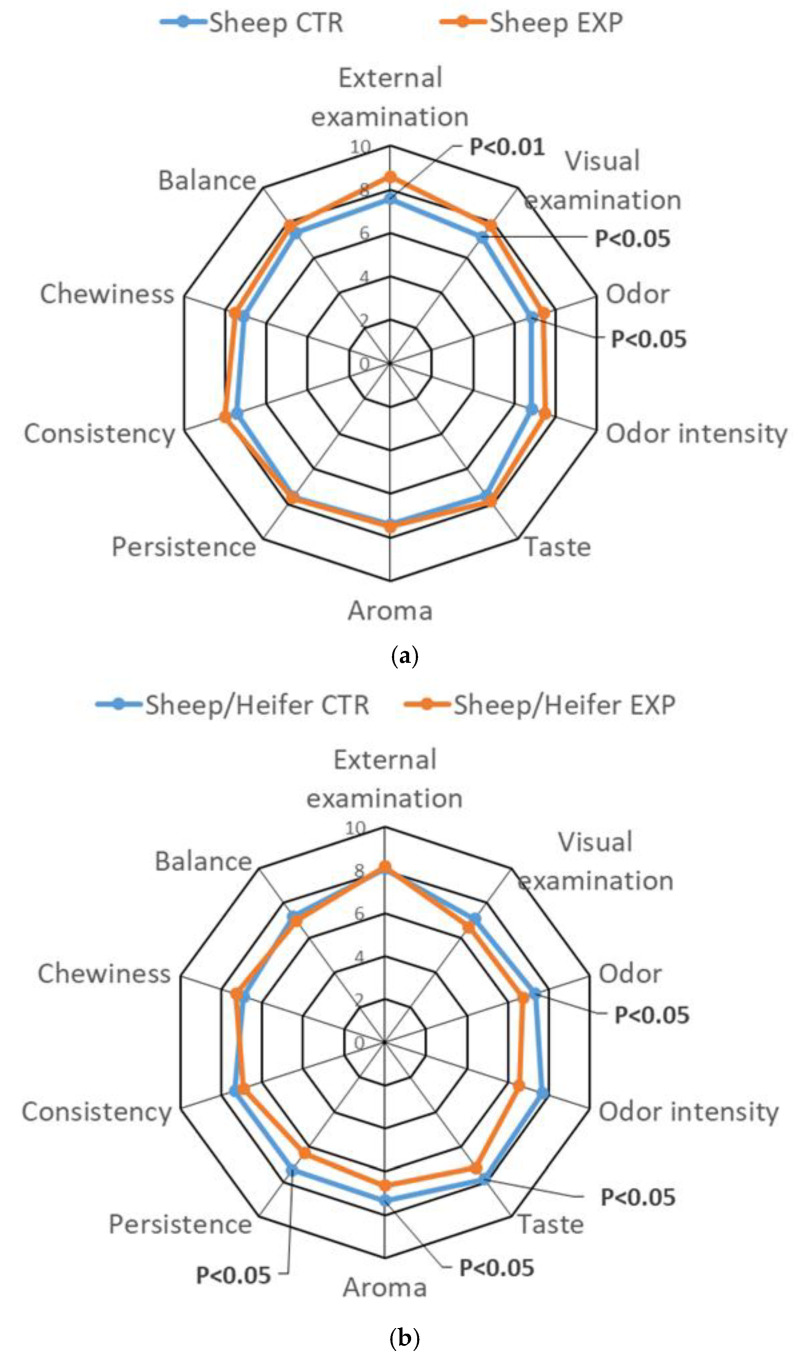

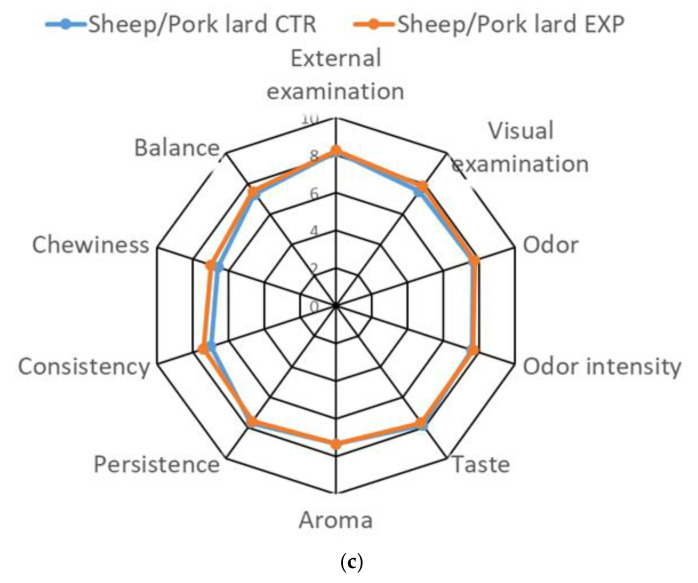
(**a**) Radar plot illustrating the sensory profile of salami produced exclusively with sheep meat (Sheep CTR vs. Sheep EXP). (**b**) Radar plot showing the sensory attributes of salami formulated with a mixture of sheep and heifer brisket (Sheep CTR/Heifer vs. Sheep EXP/Heifer). (**c**) Radar plot representing the sensory profile of salami made with sheep meat and pork lard (Sheep CTR/Pork lard vs. Sheep EXP/Pork lard).

The sensory profiles of the three salami formulations—sheep only (SM; Figure 1a), sheep/heifer mixture (HB; Figure 1b), and sheep/pork lard mixture (PB; Figure 1c)—were visualized using spider plots to highlight differences between control (CTR) and experimental (EXP) groups. These plots summarize mean panel scores for the primary sensory descriptors assessed by ONAS panelists at the end of ripening, while detailed numerical data are presented in Table 10.

Sensory evaluation indicated that inclusion of olive cake (OC) in the sheep diet produced modest but significant alterations in salami sensory characteristics. EXP salamis generally received higher scores for external appearance (*p* < 0.01), as well as for visual quality and odor intensity (*p* < 0.05). However, CTR salamis—except in the SM formulation—exhibited greater overall balance among aroma, flavor, and texture descriptors. EXP salamis, while showing a subtle herbal note, displayed slightly higher olfactory pungency and occasional oxidative sensations on the palate, attributed to feed supplementation rather than fermentative processes. Across all formulations, minor defects were noted, including excessive spicing (garlic, pepper, wine) and a soft texture with “fresh-meat” notes. The inclusion of pork backfat (PB) appeared to enhance flavor persistence and juiciness.

### 3.7. Microbial Count Evolution

**Figure 2:**
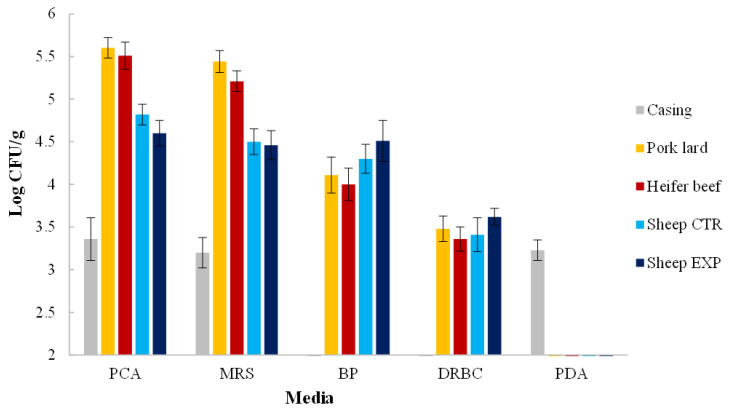
Microbial loads log CFU/g of raw materials. Results indicate mean values and standard deviation of four determinations (carried out in duplicate for two independent productions). Abbreviations: CTR = control; EXP = experimental; PCA, plate count agar for total mesophilic counts; MRS, de Man-Rogosa Sharpe agar for mesophilic LAB; BP, baird parker for staphylococci; DRBC, dichloran rose bengal chloramphenicol for yeasts; PDA, potato dextrose agar for molds.

The main microbial populations in the raw materials are presented graphically in Figure 2, with their dynamics over the 32-day salami ripening period reported in Table 11. Undesirable microorganisms, including *E. coli*, *Listeria monocytogenes*, *Salmonella* spp., coagulase-positive staphylococci (CPS), and members of the Enterobacteriaceae family, were not detected in any raw materials or salami samples at any stage of ripening; therefore, they are not included in Figure 2 or Table 11.

Natural pork casings were characterized by the presence of total mesophilic microorganisms (TMM), lactic acid bacteria (LAB), and molds at approximately 3 log CFU/g. In heifer brisket, the microbial communities were predominantly composed of LAB, with TMM and mesophilic LAB exceeding 5 log CFU/g.

Control (CTR) and experimental (EXP) sheep meat exhibited similar microbial levels irrespective of diet, with TMM and LAB reaching approximately 5 log CFU/g, and yeast populations about one log cycle lower.

As reported in Table 11, at the time of stuffing (T0), microbial populations were approximately 5 log CFU/g for total mesophilic microorganisms (TMM), lactic acid bacteria (LAB), and coagulase-negative staphylococci (CNS), while yeasts and molds were present at lower levels (~3.5 log CFU/g). After 10 days of ripening, a marked increase in TMM and LAB was observed, exceeding 7.5 log CFU/g, highlighting the pivotal role of LAB in fermentation and product acidification. Concurrently, CNS also increased significantly, reaching approximately 6.7 log CFU/g, indicating their active contribution to ripening and aroma development.

During the intermediate stage (T21), microbial populations stabilized: LAB and CNS maintained high levels (~7.7 log CFU/g), while yeasts and molds continued to increase, reaching 6.6–6.8 log CFU/g and ~6.2 log CFU/g, respectively. By the end of ripening (T32), a stable microbiological profile was established, with LAB and CNS remaining predominant at ~7.8 log CFU/g, and yeasts and molds reaching consistent levels (7.3–7.5 log CFU/g for yeasts and ~6.4 log CFU/g for molds), confirming their roles in the surface and internal microflora of the final product.

### 3.8. Characterization of Salami Microbiota

**Figure 3:**
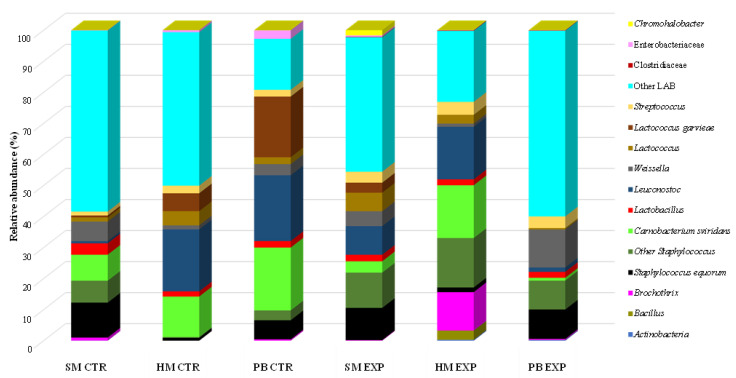
Relative abundances (%) of bacteria identified in salami at the end of ripening by Miseq Illumina. Abbreviations: SM = only sheep mixture; HM = sheep/heifer mixture; PB = sheep/pork backfat mixture; CTR = control; EXP = experimental.

Illumina sequencing technology was employed to characterize the bacterial communities present in both the experimental and control salamis at the end of ripening. Figure 3 displays the Operational Taxonomic Units (OTUs) identified with a relative abundance (RA) greater than 0.1%. The results of the classification revealed 16 taxonomic groups, primarily at the genus level. LAB communities dominated all salami production at 32 d of ripening, with a high RA % observed in PB EXP and SM CTR. The genus Staphylococcus was detected in both control and experimental salamis. The RA% of *S. equorum* in the controls varied markedly between formulations, with the highest values in SM CTR and PB CTR, and lower levels in HB CTR. In the corresponding experimental formulations, *S. equorum* remained stable or increased. All samples were characterized by the presence of *Leuconostoc* and *Weisella* with the highest RA observed in the PB EXP sample. Minor taxa such as Actinobacteria and Bacillus were sporadically detected. The lack of detection of the four pathogenic bacteria (*E. coli*, CPS, *Salmonella* spp., and *L. monocytogenes*) was observed in any salami sample.

## 4. Discussion

### 4.1. Chemical and Physical Composition of Feed

The composition of the olive cake (OC) used in the present study was consistent with previous reports employing similarly processed products [48,49]. The higher crude protein (CP) content observed in the EXP concentrate likely reflects the increased inclusion of soybean (Table 1), whereas the elevated ether extract (EE) can be attributed to residual oil in the OC, in agreement with Calabrese et al. [50]. Increased fiber fractions (NDFom and ADL) are consistent with the higher structural carbohydrate content of OC by-products, as reported in other studies [24,48,49,51]. The lignified structure of OC may reduce rumen degradability when included at levels up to 20% [52]. Despite the presumed lower digestibility of the EXP concentrate, its higher fat content compensates, resulting in estimated net energy for lactation (NEL) values comparable to the control diet, confirming the isoenergetic nature of the two concentrates.

The fatty acid profile, characterized by higher MUFA and lower PUFA content, aligns with the lipid composition of OC, which is rich in oleic acid but relatively low in linoleic (C18:2 n-6) and linolenic (C18:3 n-3) acids [53]. The increased polyphenol content reflects the phenolic compounds naturally present in OC, which exhibit antioxidant activity, reduce lipid oxidation in meat, and may modulate ruminal fermentation [50,53,54]. However, excessive dietary polyphenols can negatively affect ruminal microbiota [51].

The low dry matter content of the pasture confirms the high moisture typical of natural spring forages, while CP and fiber fractions are within the ranges reported for Sicilian pastures during the same season [55,56]. This highlights the marked variability of natural pastures, influenced by botanical composition, growth stage, and local climatic conditions [57]. The high PUFA content, mainly α-linolenic acid, underscores the nutritional value of Mediterranean herbage in promoting favorable n-3 fatty acid profiles in animal products [58]. Similarly, polyphenol concentrations support the presence of bioactive compounds typical of spontaneous Sicilian pastures, in agreement with previous findings [55,57].

However, it should be recognized that nutrient intake from pasture has not been quantified, preventing accurate calculation of total nutrient intake and assessment of the relative contribution of pasture to total feed intake. Despite this limitation, chemical and nutritional analyses remain essential for characterizing the food resource available to animals from a nutritional and bioactive perspective, especially considering that pasture was presumably used equally by animals of both groups.

### 4.2. Carcass Traits and Meat Composition

The higher hot and cold carcass weights observed in the EXP group indicate enhanced deposition of carcass mass. In addition, the trends toward a higher proportion of meat, reduced bone content, and increased separable fat are consistent with previous studies in lambs and cattle, where dietary inclusion of olive cake at low to moderate levels (15–20%) improved carcass yield and composition [24,59,60].

Colorimetric differences, including lower b* and chroma in perirenal fat and reduced b* and hue in Longissimus dorsi muscle of EXP animals, suggest a subtler meat coloration. These changes may be associated with bioactive compounds in OC by-products, such as carotenoids, and higher intramuscular fat content [59,61]. This pattern aligns with observations by Ozdogan et al. [62], who reported that OC supplementation modifies lamb meat color, particularly yellowness and hue. Moreover, other studies indicate that OC inclusion can modulate fat color and firmness, enhancing carcass acceptability without adversely affecting animal performance [24].

The lack of significant differences in shear force indicates that OC supplementation did not adversely affect meat texture, consistent with previous studies in bulls and other ruminants, where inclusion of olive cake increased intramuscular fat without compromising tenderness [24,61]. The significantly higher fat content observed in the EXP group suggests a potential lipogenic effect of OC by-products, in agreement with findings in small ruminants [63]. Correspondingly, the lower moisture content in EXP meat reflects the well-established inverse relationship between fat and water content in muscle tissue [64].

The increase in monounsaturated fatty acids (MUFA), particularly oleic acid, demonstrates that dietary supplementation with OC, which is rich in oleic acid, can favorably modify the lipid profile of meat [51]. The reduction in stearic acid (C18:0) in EXP samples may be related to enhanced Δ9-desaturase activity, as previously suggested by Chiofalo et al. [23]. Differences observed in C17:1 and C17:0 anteiso (increased in EXP) and the reduction of other C18:1 isomers likely reflect alterations in ruminal microbiota induced by the bioactive compounds, such as polyphenols and residual lipids, present in the OC by-product [22,65]. Finally, the significantly higher total fatty acid content in EXP samples corresponds to the increased lipid deposition in muscle.

### 4.3. Physical and Chemical Composition of Raw Materials Used in Salami Production

The lower pH values observed in EXP meat may be attributed to an enhanced extent of post-mortem glycolysis, likely driven by higher ante-mortem muscle glycogen reserves, which serve as the primary substrate for lactic acid production. This improved muscle energy status could be mediated by bioactive compounds, such as the polyphenols abundant in olive cake, which may influence insulin sensitivity or energy partitioning within the animal. As a result, increased glycogen deposition prior to slaughter promotes a greater post-mortem pH decline, consistent with previous feeding studies [24].

The reduced moisture content in EXP samples compared with CTR is likely a consequence of increased fat deposition, in agreement with Luciano et al. [21] and Mele et al. [22], who reported higher intramuscular fat and lower water content in lambs fed diets containing olive cake. The increase in fat content is also consistent with Chiofalo et al. [61], who demonstrated that OC inclusion enhances lipid deposition without affecting protein content. The observed reduction in ash content in EXP meat may result from a dilution effect associated with higher fat content, as minerals are predominantly concentrated in lean tissue; thus, increasing intramuscular fat proportionally reduces mineral content, as reported in lambs and other ruminants [66,67,68].

The higher polyphenol content in EXP meat reflects dietary OC supplementation, supporting the hypothesis that polyphenols can be transferred to muscle tissue, enhancing oxidative stability, as observed in previous in vivo and post-mortem studies in ruminants [21,61,69,70]. This is consistent with the greater antioxidant capacity measured in EXP meat in the present study. Correspondingly, lower peroxide values (POV) and TBARS in EXP samples further indicate that the increased antioxidant content contributes to improved oxidative stability, even in the presence of higher intramuscular fat [61,69].

The distinct lipid profiles observed among the four raw materials used for salami production reflect both species-specific characteristics and the influence of diet. The modest increases in monounsaturated fatty acids (MUFA) and oleic acid, along with the decreases in polyunsaturated fatty acids (PUFA) and linoleic acid in sheep EXP meat, are consistent with previous studies reporting the effects of OC by-products in ruminant diets [54,71]. Improvements in the n-3/n-6 ratio and slight reductions in atherogenic (AI) and thrombogenic (TI) indices suggest a nutritionally more favorable lipid profile in EXP sheep meat.

It is important to note that these results pertain to the deboned meat mixture and differ from those observed in the Longissimus dorsi (LD) muscle. The LD, after removal of intermuscular fat, represents a relatively lean muscle dominated by membrane phospholipids, which are naturally rich in n-6 PUFAs such as linoleic acid (LA) and arachidonic acid (ARA) [72]. In contrast, the deboned meat mixture comprises a heterogeneous combination of muscles and non-separable fat, including intermuscular fat, which is primarily composed of triglycerides with higher proportions of SFAs and MUFAs at the expense of PUFAs [38,72].

### 4.4. Physical and Chemical Composition of Salami

To allow for the assessment of biological variance within groups, consistent with previous study on fermented meat products [68], the experimental design included three separate processing batches within each experimental group.

The increased yellowness observed in EXP salami may be associated with the presence of tocopherols derived from the OC by-product, which, through their antioxidant activity and interaction with muscle pigments, can influence color development, particularly in the yellow component of the meat [70,73].

The reduced weight loss in EXP salami likely reflects improved moisture retention. This effect can be attributed to the lower initial water content and, more importantly, to the higher fat content, which generates a denser and more hydrophobic matrix within the dough, thereby limiting moisture reduction during drying and ripening [74]. Reduced dehydration has direct implications for texture and yield, enhancing product uniformity and minimizing the risk of excessive drying, particularly in traditional or artisanal production systems.

The observation that PB salami exhibited the lowest hardness confirms that fat type and distribution exert a greater influence on texture than the dietary regimen of the animals. The softening effect of pork fat is well-documented and is commonly used to improve the palatability of dry-cured ovine meat products, which might otherwise be perceived as overly firm or dry [15,38,68,75].

The lower moisture observed in EXP salami reflects the reduced initial water content of EXP raw meat. This trend is consistent with previous studies reporting that dry-cured sausages produced from meats with higher fat content and improved water-holding capacity experience reduced moisture losses during ripening, resulting in lower but more stable final water levels [21,24]. The final moisture content of the salamis in this study falls within the range reported for similar products [76].

Although olive cake itself is rich in minerals, the lower ash content in EXP salami can be explained by the higher fat content of these formulations, as adipose tissue contains substantially less mineral matter than lean muscle. Ash concentration is closely associated with the proportion of lean tissue and is lower in fat-rich meat products [77,78]. The reduced protein and increased fat content in EXP salami mirror the higher fat deposition observed in the carcasses and raw meat of EXP animals, consistent with other studies showing that inclusion of OC by-products in ruminant diets enhances intramuscular fat while slightly reducing protein concentration [21].

Contrary to some previous reports in which polyphenol concentration was negatively correlated with the proteolysis index (PI), no differences in PI were detected between groups. This suggests that the increased polyphenol content in EXP meat was insufficient to inhibit proteolytic activity [79]. The observed PI values were comparable to those reported for dry-fermented sausages made from sheep, beef, and pork mixtures [68,73,76]. The higher polyphenol content and increased TEAC in EXP salami correspond to the expected antioxidant contribution from OC by-products [21,24]. In line with this, the tendency for lower peroxide values (POV) and TBARS in EXP salami confirms the antioxidant effect of OC-derived polyphenols, which can delay both the initiation and propagation of lipid oxidation in meat products [20,21,24].

### 4.5. Fatty Acid Profile of Salami

The modulatory effect of the OC-supplemented diet on lipid metabolism and fatty acid desaturation is supported by the lower C16:0 and higher C18:1 c9 proportions observed in EXP salami. In particular, the SM EXP formulation, characterized by increased oleic acid (OA) and reduced saturated fatty acids (SFA), demonstrates a more favorable nutritional profile. These findings are consistent with previous studies showing that dietary supplementation with OC by-products enhances OA deposition and improves the overall lipid quality of ruminant-derived products [54,71].

The inclusion of pork backfat, which led to increased PUFA content and a reduced n-3/n-6 ratio due to elevated linoleic acid, contributed to improved softness and sensory quality of the salami [64,72]. Conversely, the addition of heifer brisket did not substantially alter the fatty acid profile compared with pure ovine formulations, indicating that the observed changes were primarily attributable to the dietary treatment rather than the species contribution [36].

The reductions in atherogenic (AI) and thrombogenic (TI) indices in SM and HB EXP salami suggest that OC supplementation in animal diets may represent an effective strategy for improving the health-related quality of fermented meat products from small ruminants.

### 4.6. Sensory Profile of Salami

The higher scores for external and visual appearance, as well as odor intensity, observed in EXP salamis indicate a more appealing color and the presence of subtle vegetal–herbaceous and underwood notes, likely associated with bioactive compounds derived from olive cake, in agreement with previous studies [24,54]. The slightly elevated olfactory pungency and occasional oxidative sensations on the palate in EXP salamis are probably linked to the dietary inclusion of unsaturated-rich OC and its influence on lipid oxidation pathways [24].

Minor defects, including excessive spicing, soft texture, and “fresh-meat” notes, observed across all formulations, were attributed to the characteristics of the raw materials and the relatively short ripening period [80]. The addition of pork backfat (PB) improved flavor persistence and juiciness, consistent with prior reports on the sensory role of monounsaturated lipids in fermented meat products [64,81].

Overall, OC supplementation imparted subtle yet distinctive sensory attributes without negatively affecting overall acceptability, suggesting that its use can contribute to a more sustainable and functionally enriched formulation of ovine salami.

### 4.7. Microbial Count Evolution by Dependent Approach

The values found for TMM, LAB, and molds in the natural pork casing were similar to those found by Pisacane et al. [82] for natural casing used in the production of traditional Italian salami. The microbial trends in heifer brisket meat are in line with those reported by Korsak et al. [83]. The similar levels of TMM, LAB, and yeasts in sheep meat, regardless of diet, are consistent with those reported by Nychas et al. [84], who observed similar levels of these microorganisms in sheep meat obtained from animals slaughtered under controlled hygienic conditions.

The high counts observed for LAB and CNS at T0 are significant because these microorganisms play a key technological role in the production of proteolytic and lipolytic enzymes, as well as in the development of aroma compounds during the ripening of salamis [85].

The increase in LAB and CNS levels after 10 days of ripening is in line with their role in spontaneous fermentation [86]. The progressive increase in yeast populations contributes to the formation of aromatic compounds and exerts competitive pressure against undesirable microorganisms [80]. The stabilization of microbial populations at T21, with the proliferation of yeasts and molds on the surface, promotes rind formation and protects the product from external contamination. The final levels of LAB and CNS are comparable to those reported in similar Italian and Portuguese fermented sausages at the end of ripening [87,88].

### 4.8. Microbial Count Evolution by Culture-Indipendent Approach

The threshold of 0.1% RA is commonly used to denote dominant bacterial taxa [89]. The dominance of LAB communities at the end of ripening aligns with previous findings reported by Busetta et al. [44].

The detection of the genus Staphylococcus is in agreement with its technological importance in fermented meats, where it contributes to aroma development and nitrate reduction [90,91]. The fact that the RA of *S. equorum* remained stable or increased in EXP formulations indicates that the dietary treatment did not negatively affect the establishment of this beneficial species.

The presence of *Leuconostoc* was observed in another study conducted on fermented dry sausages during the ripening period in Serbia and in Italy [87,92].

In fermented meats, *Weissella* spp. may contribute to early acidification, the formation of aroma compounds, and competitive exclusion of undesirable microorganisms. Their detection in the present products likely reflects natural microbial succession and suggests a transient role during the initial stages of fermentation [93] and in salami produced using Cinta Senese pork [94].

Minor taxa (for example Actinobacteria and Bacillus) did not significantly contribute to the microbial structure of either control or experimental samples.

## 5. Conclusions

Seventeen percent olive cake (OC) in the concentrate of sheep diets influenced carcass composition, meat quality, and several technological and nutritional attributes of salami produced from different formulations, including additions of heifer brisket and pork backfat. Animals receiving the OC-supplemented concentrate exhibited higher intramuscular fat and a more favorable fatty acid profile, characterized primarily by increased oleic acid and monounsaturated fatty acids (MUFA). These modifications contributed to reduced weight loss during ripening.

Moreover, dietary OC enhanced the oxidative stability and antioxidant capacity of the salami without compromising microbiological safety or negatively affecting sensory characteristics. This study suggests that integrating OC by-products into sheep diets can improve meat quality, support more sustainable production systems, and add value to animals at the end of their productive careers.

Further research is warranted to evaluate the effects of varying levels of OC supplementation on carcass traits and the technological properties of processed meat, as well as to investigate the potential of alternative ripening durations and the development of additional sheep meat-only products.

## Figures and Tables

**Table 1:** Ingredients for the formulation of the concentrates offered to ewes.

Ingredients (kg, as Feed Basis)	CTR	EXP
Partially-destoned olive cake	-	1.7
Soybean meal (48%)	1.3	1.5
Corn grains	0.5	1.7
Beet pulp	5.0	3.0
Wheat flour	3.2	2.1

CTR: control concentrate; EXP: experimental concentrate.

**Table 3:** Carcass and meat quality traits.

Items		CTR	EXP	SEM	*p* Value
Live weight		46.92	59.40	2.727	0.012
Hot carcass weight, kg		22.4	28.7	1.328	0.010
Cold carcass weight, kg		21.3	27.4	1.293	0.010
Carcass weight loss, %		5.3	4.5	0.948	0.116
Meat, %		54.9	57.8	1.074	0.083
Bone, %		34.4	28.3	1.436	0.016
Separable fat, %		10.7	13.9	0.883	0.030
Perirenal fat color	L	71.30	68.78	1.273	0.4102
	a*	2.99	1.12	0.715	0.080
	b*	11.09	7.34	1.239	0.046
	Hue	1.300	1.373	0.069	0.462
	Croma	11.90	7.49	1.206	0.019
Hot LD					
	pH	6.68	6.37	0.158	0.200
Color	L	31.65	31.69	0.737	0.965
	a*	16.24	14.73	0.577	0.081
	b*	1.57	0.90	0.170	0.012
	Hue	0.094	0.060	0.009	0.017
	Croma	16.32	14.76	0.584	0.074
Cold LD					
	pH	5.71	5.58	0.061	0.159
Color	L	36.67	36.16	0.532	0.505
	a*	20.29	20.24	0.421	0.941
	b*	3.04	2.10	0.219	0.004
	Hue	0.149	0.098	0.011	0.004
	Croma	20.53	20.34	0.422	0.758
WBS (N/cm^2^)		4.91	5.09	0.621	0.842
Water, %		76.6	74.6	0.452	0.012
Ash, %		1.05	1.04	0.014	0.819
Fat, %		2.23	3.62	0.236	0.003
Protein, %		19.7	20.6	0.337	0.129

CTR = control; EXP = experimental; LD = *Longissimus dorsi*; L = lightness; a* = redness; b* = yellowness; WBS = Warner-Bratzler shear force.

**Table 4:** Fatty acid composition of *Longissimus dorsi* (% FA tot).

Items	CTR	EXP	SEM	*p* Value
FA tot (g/100 g of DM)	8.72	12.52	1.053	0.034
C10:0	0.20	0.09	0.031	0.040
C12:0	0.43	0.28	0.084	0.244
C14:0 iso	0.23	0.14	0.051	0.240
C14:0	2.16	2.09	0.162	0.762
C14:1	0.37	0.28	0.064	0.307
C15:0 iso	0.33	0.21	0.058	0.169
C15:0	0.87	0.63	0.133	0.240
C15:1	0.33	0.30	0.017	0.275
C16:0	24.28	25.19	0.883	0.488
C16:1	1.44	1.48	0.066	0.704
C17:0 anteiso	1.38	1.65	0.065	0.019
C17:0	0.37	0.27	0.060	0.250
C17:1	0.40	0.48	0.016	0.010
C18:0	17.01	14.83	0.709	0.061
C18:1 t11 TVA	1.04	1.17	0.051	0.113
C18:1 c9 n9 OA	37.95	42.74	1.500	0.046
other C18:1	2.00	0.88	0.219	0.007
C18:2 n6 LA	4.84	3.60	0.745	0.272
C18:2 c9t11 RA	0.21	0.15	0.035	0.193
other CLA	0.42	0.59	0.099	0.253
C18:3 n6 GLA	1.17	1.04	0.147	0.538
C18:3 n3 ALA	0.39	0.46	0.054	0.354
C20:0	0.3042	0.40	0.062	0.306
C20:4 n6	1.25	0.71	0.230	0.133
C20:5 n3 EPA	0.35	0.19	0.070	0.138
C22:0	0.26	0.19	0.039	0.154
SFA	47.83	45.96	1.122	0.300
MUFA	43.53	47.32	1.099	0.039
PUFA	8.48	6.33	1.196	0.239
n3	0.74	0.65	0.080	0.459
n6	7.27	5.34	1.117	0.241
n3/n6	0.10	0.12	0.072	0.471
AI	0.64	0.63	0.036	0.890
TI	1.55	1.47	0.072	0.478

CTR = control; EXP = experimental; SEM = standard error of the means; OA = oleic acid; TVA = transvaccenic acid; LA = linoleic acid; RA = rumenic acid; CLA: Conjugated Linoleic Acids; ALA = α-linolenic acid; GLA = γ-linolenic acid; EPA = eicosapentaenoic acid; SFA: Saturated fatty acids; MUFA: Monounsaturated Fatty Acids; PUFA: Polyunsaturated Fatty Acids; AI = atherogenic index; TI = thrombogenic index.

**Table 5:** Composition of raw materials used for making salami (means ± SD).

Items	Sheep CTR	Sheep EXP	Heifer Brisket	Pork Lard
pH	5.71 ± 0.15	5.58 ± 0.12	6.10 ± 0.14	6.04 ± 0.10
Water, %	71.40 ± 0.40	67.43 ± 0.27	50.78 ± 0.42	20.66 ± 0.65
Protein, %	18.28 ± 0.29	18.58 ± 0.30	13.82 ± 0.10	6.06 ± 0.23
Fat, %	8.76 ± 0.28	13.01 ± 0.31	34.32 ± 0.21	72.63 ± 0.38
Ash, %	1.34 ± 0.17	0.98 ± 0.08	1.00 ± 0.21	0.34 ± 0.07
Polyphenols, mg GAE/g DM	1.51 ± 0.05	1.64 ± 0.11	1.05 ± 0.07	0.77 ± 0.06
TEAC, mmol Trolox/kg DM	11.42 ± 0.31	13.05 ± 0.27	6.50 ± 0.24	2.24 ± 0.14
POV, mEq O_2_/kg fat	2.50 ± 0.15	2.24 ± 0.17	1.61 ± 0.13	2.02 ± 0.23
TBARs, MDA mg/kg tissue	0.08 ± 0.01	0.06 ± 0.01	0.12 ± 0.03	0.14 ± 0.03

CTR = control; EXP = experimental; TEAC: Trolox equivalent antioxidant capacity; POV = peroxide value; TBARS = thiobarbituric acid–reactive substances; MDA = malondialdehyde.

**Table 6:** Fatty acid composition of raw materials used for salami production (% FA tot) (means ± SD).

Items	Sheep CTR	Sheep EXP	Heifer Brisket	Pork Lard
FA tot (g/100 g of DM)	27.8 ± 1.92	36.51 ± 1.94	62.22 ± 2.30	82.00 ± 4.62
C10:0	0.14 ± 0.02	0.15 ± 0.06	0.06 ± 0.01	0.10 ± 0.01
C12:0	0.10 ± 0.01	0.10 ± 0.02	0.15 ± 0.02	0.13 ± 0.02
C12:1	0.05 ± 0.01	0.03 ± 0.01	0.02 ± 0.00	0.00 ± 0.00
C14:0 iso	0.06 ± 0.01	0.06 ± 0.01	0.14 ± 0.01	0.00 ± 0.00
C14:0	3.02 ± 0.19	3.11 ± 0.20	5.71 ± 0.44	1.11 ± 0.65
C14:1	0.41 ± 0.05	0.44 ± 0.04	0.41 ± 0.05	0.01 ± 0.01
C15:0 iso	0.11 ± 0.04	0.08 ± 0.01	0.05 ± 0.00	0.03 ± 0.00
C15:0	0.12 ± 0.01	0.11 ± 0.01	0.98 ± 0.12	0.01 ± 0.00
C15:1	0.65 ± 0.04	0.68 ± 0.07	0.74 ± 0.07	0.05 ± 0.01
C16:0	25.4 ± 0.11	24.1 ± 0.91	29.7 ± 0.91	28.0 ± 0.20
C16:1	3.36 ± 0.18	3.44 ± 0.75	5.59 ± 0.50	1.58 ± 0.22
C17:0 anteiso	0.34 ± 0.11	0.36 ± 0.06	0.17 ± 0.02	0.58 ± 0.05
C17:0	1.41 ± 0.10	1.44 ± 0.11	1.15 ± 0.12	0.25 ± 0.27
C17:1	0.83 ± 0.13	0.89 ± 0.31	0.83 ± 0.08	0.19 ± 0.09
C18:0	17.6 ± 0.94	16.6 ± 1.29	15.4 ± 0.62	8.2 ± 1.18
C18:1 t11 TVA	2.58 ± 0.25	2.83 ± 0.28	3.30 ± 0.13	0.00 ± 0.00
C18:1 c9 n9 OA	34.2 ± 1.08	35.7 ± 2.07	28.2 ± 2.18	39.8 ± 1.12
other C18:1	3.37 ± 0.15	3.42 ± 0.28	3.11 ± 0.28	2.25 ± 0.35
other C18:2	1.04 ± 0.34	1.14 ± 0.24	0.79 ± 0.29	0.03 ± 0.01
C18:2 n6 LA	3.19 ± 0.37	2.02 ± 0.36	1.11 ± 0.45	19.76 ± 0.97
C18:2 c9t11 RA	0.48 ± 0.05	0.44 ± 0.03	0.69 ± 0.06	0.04 ± 0.00
other CLA	0.14 ± 0.13	0.14 ± 0.11	0.11 ± 0.05	0.02 ± 0.00
C18:3 n6 GLA	0.04 ± 0.01	0.03 ± 0.01	0.02 ± 0.00	0.02 ± 0.01
C18:3 n3 ALA	0.76 ± 0.02	0.78 ± 0.01	0.26 ± 0.01	1.86 ± 0.02
C20:0	0.08 ± 0.30	0.08 ± 0.03	0.08 ± 0.04	0.12 ± 0.05
C20:4 n6	0.19 ± 0.02	0.30 ± 0.08	0.07 ± 0.01	0.16 ± 0.05
C20:5 n3 EPA	0.11 ± 0.02	0.10 ± 0.05	0.00 ± 0.01	0.03 ± 0.00
C22:0	0.09 ± 0.06	0.09 ± 0.07	0.01 ± 0.00	0.02 ± 0.00
SFA	48.2 ± 0.98	45.9 ± 1.29	53.4 ± 1.23	37.9 ± 1.21
MUFA	45.4 ± 2.39	47.5 ± 2.38	42.2 ± 2.74	43.9 ± 2.16
PUFA	5.94 ± 0.65	4.94 ± 0.46	3.06 ± 0.54	21.9 ± 0.53
n3	0.87 ± 0.02	0.88 ± 0.09	0.27 ± 0.01	1.89 ± 0.07
n6	3.42 ± 0.36	2.35 ± 0.35	1.20 ± 0.48	19.9 ± 0.87
n3/n6	0.25 ± 0.06	0.38 ± 0.05	0.22 ± 0.06	0.09 ± 0.01
AI	0.76 ± 0.05	0.72 ± 0.02	1.21 ± 0.07	0.50 ± 0.03
TI	1.69 ± 0.05	1.57 ± 0.08	2.24 ± 0.13	0.99 ± 0.07

CTR = control; EXP = experimental; FA = Fatty acid; OA = oleic acid; TVA = transvaccenic acid; LA = linoleic acid; RA = rumenic acid; ALA = α-linolenic acid; CLA: Conjugated Linoleic Acids; GLA = γ-linolenic acid; EPA = eicosapentaenoic acid; SFA: Saturated fatty acids; MUFA: Monounsaturated Fatty Acids; PUFA: Polyunsaturated Fatty Acids; AI = atherogenic index; TI = thrombogenic index.

**Table 7:** Physical parameters of salami at end of ripening.

Mixture (M)	SM		HB		PB		SEM	*p* Value		
Group (G)	CTR	EXP	CTR	EXP	CTR	EXP		G	M	G*M
L*	41.0	43.0	43.3	44.0	49.4	49.1	3.656	0.790	0.143	0.949
a*	14.0	14.5	12.0	13.5	12.9	13.3	1.136	0.367	0.393	0.864
b*	5.90	6.47	4.17	4.82	5.79	5.65	0.431	0.315	0.003	0.600
Hue	0.40	0.43	0.34	0.35	0.42	0.40	0.031	0.808	0.040	0.693
Croma	15.2	15.9	12.6	14.3	14.1	14.5	1.133	0.323	0.213	0.832
Weight loss, %	44.1 ^a^	41.4 ^b^	44.4 ^a^	39.5 ^b^	41.7 ^a^	37.4 ^b^	1.039	0.001	0.025	0.039
pH	5.88	5.72	5.94	5.64	5.76	5.54	0.099	0.016	0.612	0.047
Hardness, N/mm^2^	0.67	0.65	0.58	0.56	0.55	0.55	0.013	0.266	<0.001	0.606

The results indicate mean values of 3 measurements performed on each sample. SM = only sheep mixture; HB = sheep/heifer mixture; PB = sheep/pork backfat mixture; CTR = control; EXP = experimental; SEM = standard error of the means; L* = lightness; a* = redness; b* = yellowness. On horizontal rows: a and b = *p* ≤ 0.05 in the comparison between groups within the same mixture.

**Table 8:** Chemical parameters of salami at end of ripening.

Mixture (M)	SM		HB		PB		SEM	*p* Value		
Group (G)	CTR	EXP	CTR	EXP	CTR	EXP		G	M	G*M
Water, %	48.1 ^A^	40.1 ^B^	44.4 ^A^	39.3 ^B^	40.5 ^A^	36.6 ^B^	0.338	<0.001	<0.001	0.001
Ash, %	6.71 ^A^	5.92 ^B^	6.56 ^A^	5.46 ^B^	5.89	5.91	0.070	<0.001	0.002	<0.001
Protein, %	31.2 ^A^	26.3 ^B^	28.1 ^A^	24.7 ^B^	26.5	26.3	0.315	<0.001	<0.001	<0.001
Fat, %	13.9 ^B^	27.6 ^A^	20.7 ^B^	30.3 ^A^	27.1 ^B^	31.0 ^A^	0.074	<0.001	<0.001	<0.001
PI %	12.7	12.3	12.1	12.0	11.9	12.0	0.159	0.289	0.085	0.316
Polyphenols, mg GAE/g DM	1.31 ^A^	1.59 ^B^	1.21	1.25	0.92	0.96	0.007	0.158	0.046	0.003
TEAC, mmol Trolox/kg DM	10.60 ^b^	11.98 ^a^	8.13	8.60	5.09	5.35	0.076	0.219	0.017	0.046
POV, mEq O_2_/kg fat	1.75	1.70	2.42	2.34	2.92	2.65	0.093	<0.001	<0.001	<0.001
TBARS, mg MDA/kg	0.28	0.25	0.33	0.28	0.31	0.026	0.011	<0.001	0.073	0.128

The results indicate mean values of 3 measurements performed on each sample. SM = only sheep mixture; HB = sheep/heifer mixture; PB = sheep/pork backfat mixture; CTR = control; EXP = experimental; SEM = standard error of the means; PI = Proteolytic index (NPN/TN ×100); GAE = gallic acid equivalent; TEAC = Trolox equivalent antioxidant capacity; POV = peroxide value; TBARS = thiobarbituric acid–reactive substances; MDA = malondialdehyde. On horizontal rows: a and b = *p* ≤ 0.05, A and B = *p* ≤ 0.01 in the comparison between groups within the same mixture.

**Table 9:** Fatty acid composition of salami at end of ripening (% FA tot).

Mixture (M)	SM		HB		PB		SEM	*p* Value		
Group (G)	CTR	EXP	CTR	EXP	CTR	EXP		G	M	G*M
FA tot (g/100 g of DM)	12.5 ^B^	20.6 ^A^	18.6 ^B^	27.0 ^A^	23.91 ^B^	26.9 ^A^	1.344	<0.001	<0.001	0.001
C10:0	0.15	0.16	0.12	0.13	0.14	0.15	0.009	0.462	0.086	0.277
C12:0	0.09	0.10	0.10	0.10	0.11	0.10	0.007	0.997	0.179	0.218
C12:1	0.05	0.05	0.04	0.04	0.03 ^B^	0.04 ^A^	0.003	0.0023	<0.001	0.003
C14:0 iso	0.07	0.06	0.07	0.07	0.03 ^B^	0.05 ^A^	0.004	0.036	<0.001	0.007
C14:0	3.25	3.03	3.28	3.67	2.56	2.73	0.133	0.311	<0.001	0.091
C14:1	0.47	0.52	0.45	0.47	0.26	0.38	0.026	0.009	<0.001	0.127
C15:0 iso	0.13	0.14	0.10	0.11	0.08	0.11	0.007	0.009	<0.001	0.131
C15:0	0.10	0.10	0.28 ^A^	0.23 ^B^	0.07	0.09	0.008	0.089	<0.001	0.001
C15:1	0.75	0.72	0.67	0.71	0.47	0.54	0.053	0.496	0.001	0.607
C16:0	25.0 ^A^	23.0 ^B^	25.9	25.1	26.1	25.3	0.511	<0.001	<0.001	0.002
C16:1	3.38	3.29	3.77	3.85	2.94	3.24	0.152	0.436	0.001	0.453
C17:0 anteiso	0.36	0.33	0.28	0.34	0.39	0.41	0.020	0.374	0.002	0.123
C17:0	1.67 ^a^	1.48 ^b^	1.39 ^b^	1.50 ^a^	1.00	1.19	0.061	0.484	<0.0001	0.014
C17:1	0.95	0.87	0.80	0.95	0.66	0.78	0.048	0.123	0.002	0.059
C18:0	19.4	17.2	18.0	16.4	14.3	15.1	0.811	0.153	0.001	0.187
C18:1 t11 TVA	2.89	3.09	2.87	2.93	1.75	2.21	0.106	0.012	<0.0001	0.207
C18:1 c9 n9 OA	33.8 ^b^	35.6 ^a^	32.1 ^b^	34.5 ^a^	36.5	36.7	0.421	0.127	0.027	0.043
other C18:1	3.39	3.57	3.61	3.30	2.98	3.23	0.144	0.697	0.029	0.134
other C18:2	1.20	1.21	1.01	1.21	0.76	0.84	0.076	0.139	0.0001	0.470
C18:2 n6 LA	2.70 ^a^	1.89 ^b^	2.06	1.81	6.36 ^A^	4.00 ^B^	0.231	0.005	<0.0001	<0.0001
C18:2 c9 t11 RA	0.44	0.43	0.46	0.49	0.29	0.37	0.002	<0.0001	<0.0001	<0.0001
other CLA	0.18	0.17	0.16	0.14	0.05	0.05	0.050	0.751	0.673	0.999
C18:3 n6 GLA	0.03	0.04	0.03	0.03	0.03	0.03	0.003	0.023	0.002	0.112
C18:3 n3 ALA	0.72	0.78	0.68	0.71	0.92 ^A^	0.85 ^B^	0.005	0.001	<0.0001	<0.0001
C20:0	0.14	0.12	0.11	0.06	0.10	0.06	0.061	0.470	0.705	0.975
C20:4 n6	0.12	0.32	0.21	0.12	0.12	0.09	0.009	0.001	<0.0001	<0.0001
C20:5 n3 EPA	0.05	0.06	0.07	0.05	0.21	0.12	0.004	<0.0001	<0.0001	<0.0001
C22:0	0.07	0.12	0.07	0.06	0.05	0.05	0.028	0.571	0.280	0.552
SFA	50.4 ^A^	45.9 ^B^	49.7 ^a^	47.7 ^b^	45.0	44.9	0.911	0.008	0.001	0.039
MUFA	45.6 ^b^	47.9 ^a^	44.3 ^b^	46.9 ^a^	45.6	46.1	0.720	0.041	0.054	0.019
PUFA	5.64	4.98	4.68	4.57	8.83 ^A^	6.42 ^B^	0.268	0.054	<0.0001	<0.0001
n3	0.77	0.83	0.74	0.75	1.13 ^A^	0.97 ^B^	0.016	0.773	<0.0001	<0.0001
n6	3.06 ^A^	2.15 ^B^	2.30	1.96	6.51 ^A^	4.12 ^B^	0.230	0.007	<0.0001	<0.0001
n3/n6	0.29	0.36	0.33	0.39	0.17	0.24	0.030	0.562	<0.0001	0.043
AI	0.76 ^a^	0.66 ^b^	0.80	0.78	0.67	0.67	0.020	0.025	<0.0001	0.047
TI	1.79 ^A^	1.52 ^B^	1.83 ^a^	1.67 ^b^	1.45	1.49	0.049	0.004	<0.0001	0.019

The results indicate mean values of 3 measurements performed on each sample. SM = only sheep mixture; HB = sheep/heifer mixture; PB = sheep/pork backfat mixture; CTR = control; EXP = experimental; SEM = standard error of the means; OA = oleic acid; TVA = transvaccenic acid; LA = linoleic acid; RA = rumenic acid; ALA = α-linolenic acid; GLA = γ-linolenic acid; DGLA = diomo-γ-linolenic acid; EPA = eicosapentaenoic acid; AI = atherogenic index; TI = thrombogenic index. On horizontal rows: a and b = *p* ≤ 0.05, A and B = *p* ≤ 0.01 in the comparison between groups within the same mixture.

**Table 10:** Sensorial profile of salami at end of ripening.

Mixture (M)	SM		HB		PB		SEM	*p* Value		
Group (G)	CTR	EXP	CTR	EXP	CTR	EXP		G	M	G*M
External examination	7.58 ^B^	8.58 ^A^	8.08	8.18	8.17	8.25	0.196	0.016	0.800	0.032
Visual examination	7.17 ^b^	7.83 ^a^	7.08	6.64	7.50	7.83	0.200	0.264	0.000	0.022
Odor	6.83 ^b^	7.42 ^a^	7.33 ^a^	6.73 ^b^	7.67	7.75	0.230	0.902	0.008	0.040
Intensity of odor	6.83	7.50	7.67	6.55	7.58	7.67	0.258	0.571	0.097	0.003
Taste	7.50	7.83	7.83 ^a^	7.18 ^b^	7.83	7.67	0.196	0.321	0.452	0050
Aroma	7.42	7.50	7.33 ^a^	6.64 ^b^	7.33	7.33	0.259	0.268	0.172	0.039
Flavor persistence	7.58	7.67	7.33 ^a^	6.36 ^b^	7.67	7.58	0.294	0.182	0.162	0.012
Texture	7.42	8.00	7.33	6.91	7.00	7.42	0.240	0.327	0.035	0.088
Chewiness	7.08	7.50	6.92	7.27	6.58	7.00	0.253	0.058	0.143	0.286
Sensory balance	7.42	7.83	7.25	7.00	7.33	7.50	0.239	0.537	0.115	0.254

SM = only sheep mixture; HB = sheep/heifer mixture; PB = sheep/pork backfat mixture; CTR = control; EXP = experimental; SEM = standard error of the means. On horizontal rows: a and b = *p* ≤ 0.05, A and B = *p* ≤ 0.01 in the comparison between groups within the same mixture.

**Table 11:** Microbiological count of salami during of ripening.

Mixture (M)	Ripening Time Days	SM		HB		PB		SEM	*p* Value		
Group (G)		CTR	EXP	CTR	EXP	CTR	EXP		G	M	G*M
PCA	0	4.87	4.78	4.95	5.08	5.09	5.05	0.107	0.985	0.079	0.558
	10	7.84	7.73	7.71	7.61	7.83	7.88	0.127	0.602	0.335	0.785
	21	7.91	7.88	7.83	7.81	7.93	7.88	0.099	0.684	0.638	0.988
	32	7.91	7.84	7.89	7.86	7.90	7.93	0.089	0.769	0.871	0.847
MRS	0	4.58	4.55	4.53	4.88	4.78	4.65	0.134	0.570	0.478	0.196
	10	7.65	7.45	7.60	7.50	7.64	7.52	0.113	0.139	0.956	0.880
	21	7.72	7.70	7.65	7.67	7.70	7.78	0.073	0.638	0.563	0.800
	32	7.84	7.78	7.74	7.79	7.83	7.89	0.067	0.765	0.389	0.622
BP	0	4.71	4.60	4.63	4.55	4.77	4.70	0.107	0.334	0.415	0.981
	10	6.67	6.77	6.57	6.52	6.43	6.78	0.131	0.228	0.414	0.327
	21	6.54	6.73	6.81	6.78	6.30	6.65	0.081	0.020	0.004	0.092
	32	7.74	7.69	7.78	7.84	7.68	7.55	0.071	0.497	0.042	0.419
DRBC	0	3.79	3.55	3.68	3.61	3.51	3.49	0.101	0.201	0.222	0.536
	10	5.54	5.50	5.38	5.66	5.28	5.49	0.130	0.174	0.500	0.448
	21	6.58	6.79	6.74	6.76	6.48	6.65	0.097	0.109	0.182	0.595
	32	7.33	7.43	7.50	7.59	7.37	7.32	0.101	0.577	0.134	0.712
PDA	0	3.45	3.38	3.39	3.59	3.69	3.33	0.107	0.392	0.653	0.055
	10	5.38	5.21	5.39	5.23	5.53	5.18	0.110	0.021	0.852	0.631
	21	6.19	6.37	6.01	6.19	6.25	6.21	0.098	0.198	0.192	0.446
	32	6.39	6.42	6.50	6.58	6.35	6.34	0.099	0.684	0.159	0.902

Results are expressed as log CFU/g and indicate mean values of four plate counts (carried out in duplicate for two independent productions). Abbreviations: SEM, standard error of mean; SM = only sheep mixture; HB = sheep/heifer mixture; PB = sheep/pork backfat mixture; CTR = control; EXP = experimental; PCA, plate count agar for total mesophilic counts; MRS, de Man-Rogosa Sharpe agar for mesophilic LAB; BP, baird parker for staphylococci; DRBC, dichloran rose bengal chloramphenicol for yeasts; PDA, potato dextrose agar for moulds.

## Data Availability

The datasets used and/or analysed during the current study are available from the corresponding author on reasonable request.
